# Distribution, Interaction and Functional Profiles of Epiphytic Bacterial Communities from the Rocky Intertidal Seaweeds, South Africa

**DOI:** 10.1038/s41598-019-56269-2

**Published:** 2019-12-27

**Authors:** Ramganesh Selvarajan, Timothy Sibanda, Siddarthan Venkatachalam, Henry J. O. Ogola, Chinedu Christopher Obieze, Titus A. Msagati

**Affiliations:** 1 Department of Environmental Sciences, College of Agricultural and Environmental Sciences, UNISA, Johannesburg, South Africa; 2 Department of Biological Sciences, University of Namibia, Mandume Ndemufayo Ave, Pionierspark, Windhoek, Namibia; 3 Arctic Division, National Centre for Polar and Ocean Research, Vasco-da-Gama, Goa, India; 4 Centre for Research, Innovation and Technology, Jaramogi Oginga Odinga University of Science and Technology, Bondo, Kenya; 5 Department of Microbiology, University of Port Harcourt, Port Harcourt, Nigeria; 6 Nanotechnology and Water Sustainability Research Unit, College of Science, Engineering and Technology, University of South Africa-Science Campus, Florida, South Africa

**Keywords:** Molecular ecology, Ecological networks, Microbial ecology

## Abstract

Interrelations between epiphytic bacteria and macroalgae are multifaceted and complicated, though little is known about the community structure, interaction and functions of those epiphytic bacteria. This study comprehensively characterized the epiphytic bacterial communities associated with eight different common seaweeds collected from a rocky intertidal zone on the Indian Ocean at Cape Vidal, South Africa. High-throughput sequencing analyses indicated that seaweed-associated bacterial communities were dominated by the phyla *Proteobacteria*, *Bacteroidetes*, *Firmicutes*, *Cyanobacteria*, *Planctomycetes*, *Actinobacteria* and *Verrucomicrobia*. Energy-dispersive X-ray (EDX) analysis showed the presence of elemental composition in the surface of examined seaweeds, in varying concentrations. Cluster analysis showed that bacterial communities of brown seaweeds (SW2 and SW4) were closely resembled those of green seaweeds (SW1) and red seaweeds (SW7) while those of brown seaweeds formed a separate branch. Predicted functional capabilities of epiphytic bacteria using PICRUSt analysis revealed abundance of genes related to metabolic and biosynthetic activities. Further important identified functional interactions included genes for bacterial chemotaxis, which could be responsible for the observed association and network of elemental-microbes interaction. The study concludes that the diversity of epiphytic bacteria on seaweed surfaces is greatly influenced by algal organic exudates as well as elemental deposits on their surfaces, which triggers chemotaxis responses from epiphytic bacteria with the requisite genes to metabolise those substrates.

## Introduction

Microorganisms are found associated with a wide variety of hosts including animate and inanimate^1^. In some cases, microorganisms are increasingly recognized to play a significant role in host health and metabolism^2,3^. For example, in terrestrial ecosystem, plants are highly dependent on their associated microbes to support their development and protection under stressors^4^. Similarly, in marine ecosystems, the surface of marine organisms like macroalgae (seaweeds) often represents a highly active association between hosts and microbes^5^. These microbes play crucial roles such as supplying the seaweeds with vitamins^6^ and fatty acids^7^, as well as regulating growth of the seaweed^8^. In addition, some are producers of antimicrobials^9^ while others are involved in nutrient biogeochemical cycling and hence support growth and adaptation and acclimation to environmental stress^10^. Seaweed-associated epiphytic microbial communities are extremely complex, and can best be described as cosmopolitan, constituting of bacteria, fungi, archaea, viruses diatoms and protozoa^8^. However, their biodiversity and functional associations remain largely unexplored and poorly understood.

Earlier, studies investigated the bacterial communities of seaweeds using culture dependent analysis^7,11–13^. In one such study, Beleneva and Zhukova^14^ reported *Vibrio* spp. as the single most abundant bacteria on the surfaces of brown and red macroalgal species collected from Peter the Great Bay in the Sea of Japan. Other identified bacterial strains included *Sulfitobacter* spp., *Halomonas* spp., *Acinetobacter* spp., *Planococcus* spp., *Arthrobacter* spp., and *Agromyces* spp. Similarly, Wang *et al*.^15^ reported *Halomonas* and *Vibrio* as the major bacterial groups isolated from the surfaces of four different seaweed in China. They observed closely-knit genetic relationships among the isolates, which also displayed host species specificity. Host specificity observations as well as temporal and spatial variations were further confirmed by culture-independent studies of Egan *et al*.^16^. Other researchers studied bacterial communities in green (*Chlorophyta*), brown (*Phaeophyta*) and red (*Rhodophyta*) macroalgae using community fingerprinting methods such as denaturing gradient gel electrophoresis (DGGE) and terminal restriction fragment length polymorphism (T-RFLP)^17–19^. Most community analysis revealed that members of *Alphaproteobacteria* and *Gammaproteobacteria* were the dominant bacterial-seaweed associated phyla. Other frequently encountered phyla include the *Bacteroidetes, Actinobacteria, Planctomycetes* and *Chloroflexi*^19–22^. The advent of culture independent techniques has seen a great deal of research effort being focused on exploring microbial communities associated with seaweeds in order to understand the ecological basis of seaweed-microbe interactions^8^.

Some research findings seem to insinuate that different macroalgal species in the same ecological niche are associated with specific bacterial communities^19,20^, while others suggest that macroalgal species can have similar bacterial communities even if they are located in different ecological niches^14,15^. In addition to spatial variability, epiphytic bacterial community compositions are also driven by differences in seasonal, environmental and physiological characteristics including elemental composition and nutrient availability^23^. The occurrence and relative abundance of epiphytic bacterial species may also vary depending on their specific interactions with abiotic factors.

In the last two decades, advances in next generation sequencing technologies have now made it feasible to characterize the core seaweed-associated epiphytic bacterial communities from different ecological niches. Up until now, the diversity of epiphytic bacterial communities has most likely been underestimated due to the limitations of the conventional methods^8,16^. Consequently, there is a need for in-depth analysis to characterize the epiphytic bacterial community compositions as well as to understand the epiphytic bacterial functions and their interactions with abiotic gradients. In this study, we used next-generation sequencing analysis to provide in-depth insights into the diversity of seaweed-associated epiphytic bacterial communities of eight different seaweed species that are commonly found in the rocky intertidal zone at Cape Vidal, South Africa. Additionally, prediction of functional abilities and bacteria-seaweed interactions using co-network analysis allowed us to infer differences and relationships across seaweed epiphytes. To the best of our knowledge, this is the first study to investigate the seaweed associated epiphytic bacterial communities using next generation sequencing technology in South Africa.

## Results

**Figure 1:**
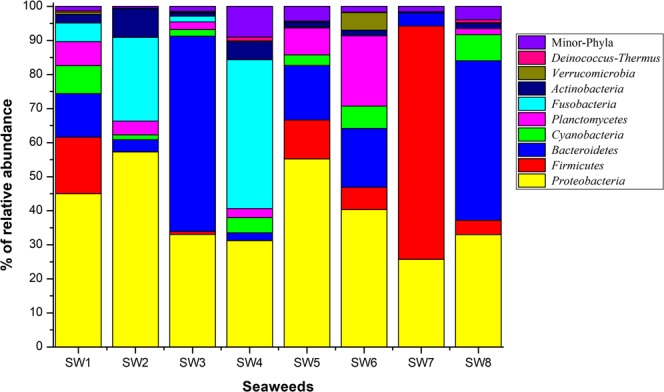
Relative abundance of sequences representing bacterial phyla constituting epiphytic bacterial communities on different seaweed samples.

Scanning electron microscopy of collected seaweeds revealed variations in surface morphology between the seaweed species (S. Fig. 1). Results of energy-dispersive X-ray (EDX) analysis showed the presence of ten different chemical elements in the cell wall at varying concentrations dependent on seaweed type. Among the identified ten elements, the most abundant was carbon, with higher level detected in *Hypnea rosea* (SW3: 71.01%), followed by *Sargassum incisifolium* (SW2: 64.14%). Comparatively, red seaweeds (SW6 & SW7) had higher levels of calcium than other seaweeds, while potassium was higher in green and brown seaweeds than red seaweeds. Sulphur content ranged between 0.36–4.45%, with a magnitude in the order of SW1≫SW5 > SW8 > SW6 > SW3 > SW7 > SW2 > SW4. The detailed elemental compositions in the seaweeds are given in S. Table 1.

### Epiphytic bacterial diversity

**Figure 2:**
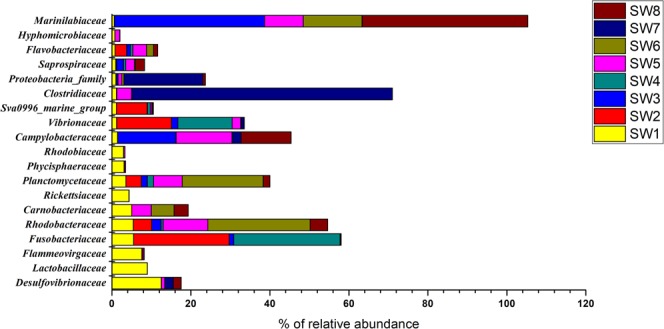
Relative abundance and distribution of epiphytic bacterial families on seaweed samples.

**Figure 3:**
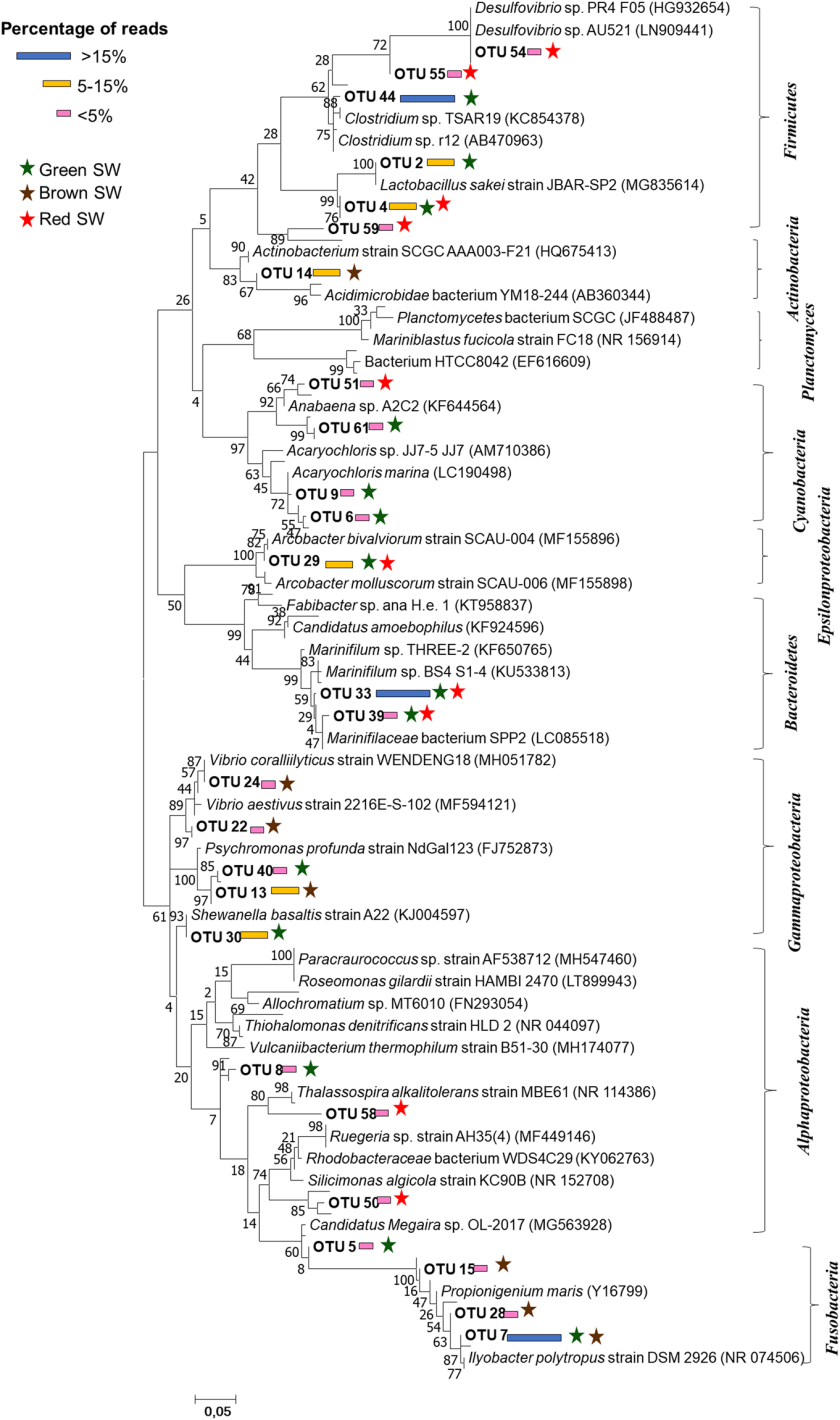
Maximum-Likelihood phylogenetic tree showing the 16S rRNA gene sequences of top 10 OTUs observed in the eight different seaweeds. Bootstrap values are given in percentage at branch nodes based on 1000 resembling. The scale bar indicates evolutionary distance.

After filtering out low quality and chimeric sequences, a total of 243,488 quality reads were obtained from the eight different seaweed samples, which were then used for downstream analysis. Overall, 4,352 OTUs were predicted across all the samples based on the Silva rRNA gene database at the cut-off level of 97%. Among the eight seaweed samples, brown seaweed SW2 (1,038 OTUs) harboured the highest number of unique OTUs, while the lowest number of OTUs were recorded in red seaweed SW7 (120 OTUs). In contrast, sample SW8 recorded the highest OTUs (746 OTUs) among the green seaweeds. The calculated alpha diversity indices including the Shannon and Simpson diversity are presented in Table 1. The Chao1 index, considered as expected OTU richness estimator, showed the lowest number of OTUs richness in SW7 (83.11) and the highest in SW8 sample (1,072.71). Similarly, the diversity index (Shannon-H index) was highest in SW5 (4.847) and lowest in SW7 (1.544). There was no significant difference between the bacterial species richness (Chao_1) and the bacterial diversity (Shannon_H) across all the samples. To test the similarity between the seaweed samples, cluster analysis of the samples was performed and a cluster tree was constructed (S. Fig. 2). Cluster analysis showed that bacterial communities of brown seaweeds (SW2 and SW4) were phylogenetically closer to those of green seaweeds (SW1) and red seaweeds (SW7) while those of brown seaweeds formed a separate clade. Further, the PCoA plot explained 53% of the observed variation, with the first axis explaining 29% and the second axis explaining 24% of the variation respectively (S. Fig. 3). Similar to cluster analysis results, PCoA analysis based on Bray-Curtis similarities confirmed that brown seaweeds SW2 and SW4 were strongly clustered together and closer to SW1; whereas red seaweed (SW3, SW6) were grouped together with green seaweed (SW8) respectively.

**Table 1:** Estimated OTU richness and diversity indices of the eight different seaweeds collected from intertidal zones of Mission Rocks, South Africa.

	SW1	SW2	SW3	SW4	SW5	SW6	SW7	SW8
OTUs	431	1038	504	316	737	460	120	746
Dominance_D	0.034	0.052	0.121	0.147	0.025	0.045	0.431	0.127
Simpson_1-D	0.967	0.948	0.879	0.853	0.975	0.955	0.569	0.873
Shannon_H	4.502	4.065	3.075	3.024	4.847	3.983	1.544	3.647
Evenness_e^H/S	0.209	0.0561	0.043	0.065	0.1728	0.117	0.039	0.051
Chao-1	525.01	523.13	611.85	480.19	997.01	722.62	83.11	1072.71
ACE	220.07	179.33	381.61	173.42	444.1	307.72	40.38	445.39

Phylogenetic classification revealed 24 bacterial phyla distributed across all seaweed samples. The distribution of the bacterial phyla for the eight seaweeds is given in Fig. 1. The three most dominant phyla were *Proteobacteria* with a relative abundance of between 25.69% of the total 16S rRNA gene sequences recorded in SW7 and 57.25% of those recorded in SW2, *Bacteroidetes* (2.29% in SW4 to 57.36% in SW3) and *Firmicutes* (0.03% in SW4 to 68.4% in SW7). Other major phyla including *Cyanobacteria* (0.18% in SW3 to 8.22% in SW8), *Planctomycetes* (0.04% in SW8 to 20.64% in SW6), *Actinobacteria* (0.02% in SW3 to 8.46% in SW2) and *Verrucomicrobia* (0.02% in SW1 to 5.17% in SW6) were also identified among all seaweed samples. Phylum *Fusobacteria* was not recorded in sample SW8 though it was recorded in other samples at relative abundances ranging from 0.04 to 43.70%. Statistical analysis revealed that with the exception of the phylum *Fusobacteria* (P = 0.0074), other phyla did not differ significantly in their relative abundances. Low frequency sequences belonging to some minor phyla were also found (S. Table 2).

Family level taxonomic classification of the epiphytic bacterial communities showed that a total of 225 families were present across all seaweed samples. However, the distribution and relative abundances epiphytic bacterial communities was dependent on seaweed types. Family *Fusobacteriaceae* was dominant in both brown seaweeds, with a relative abundance of 24.17% and 26.89% for SW2 and SW4, respectively. The next most dominant family was *Psychromonadaceae* (18.27% in SW2 and 0.09% in SW4), followed by *Vibrionaceae* (13.85% in SW2 and 13.73% in SW4). Sequences representing the families *Campylobacteraceae, Desulfovibrionaceae*, *Lactobacillaceae, Rhodobacteraceae, Carnobacteriaceae, Marinifilaceae*, and *Clostridiaceae* were highly associated with red and green seaweeds. For example, sequences representing the family *Campylobacteraceae* were high in green seaweed SW5 (14.11%) and SW8 (12.64%), while the family *Rhodobacteraceae* was dominant in red seaweed SW6 (25.86%). Another dominant family was *Marinilabiaceae*, whose relative abundance was high in red seaweed SW3 (38.01%) and green seaweed SW8 (41.90%) respectively. Figure 2 details the distribution of epiphytic bacterial families on seaweed samples.

**Figure 4:**
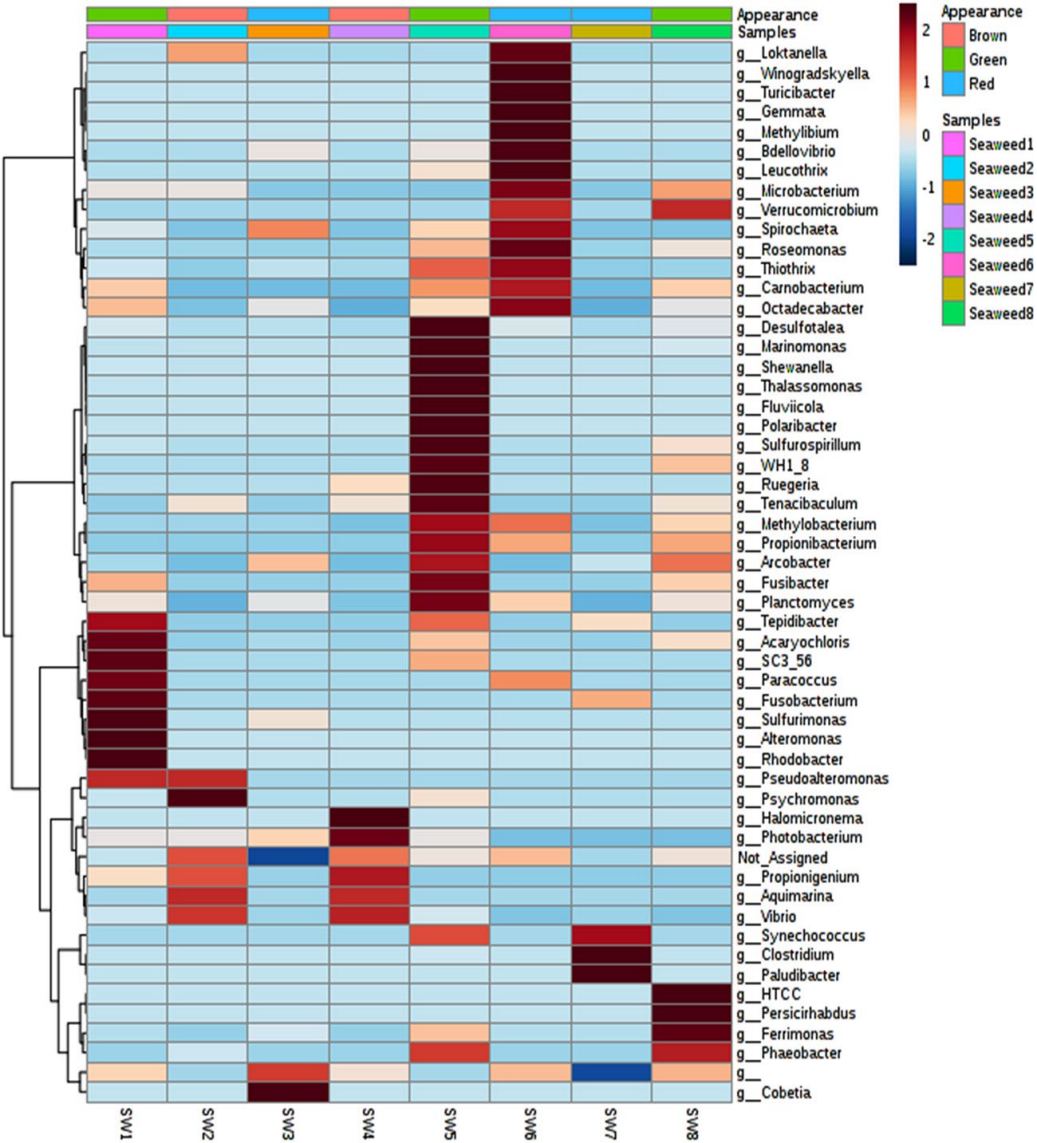
Genus level distribution of epiphytic bacterial communities on different seaweed leaf samples.

In depth analysis of top 10 OTUs per sample revealed of 49 dominant OTUs across the eight seaweed samples. This data was used to construct a phylogenetic tree (Fig. 3). Results showed that epiphytic bacteria on brown seaweed samples (SW2 and SW4) were closely related to *Propionigenium* sp. (OTU 7, 15, 28), *Psychromonas* sp. (OTU 13), *Acidomicrobium* sp. (OTU 14) and *Vibrio* sp. (OTU 22, 24). In contrast, bacterial communities on green seaweeds were closely related to *Lactobacillus* sp. (OTU 2, 4), *Candidatus_Amoebophilus* (OTU 5), *Ilyobacter* sp. (OTU 7), *Acaryochloris* sp. (OTU 6, 9) and *Thallossospira* sp. (OTU 8). However, subtle OTUs distribution differences was observed within the green seaweeds. For example, bacterial sequences closely resembling those of *Clostridium* sp. (OTU 44), *Arcobacter* sp. (OTU 28), *Marinifilum* sp. (OTU 33) and *Shewanella* sp. (OTU 30) were dominant in SW5, while those of *Anabaena* sp. (OTU 61) and members of *Marinifilum* and *Arcobacte*r spp. were dominant in SW8. Lastly, the red seaweeds were dominated with sequences closely matching those of *Desulfovibrio* sp. (OTU 54, 55), *Actinobacterium* sp. (OTU 59) and members *Marinifilum* and *Arcobacter* spp. respectively. The distribution of the core epiphytic bacterial genera (>5% abundance) across the eight different seaweeds samples is shown in a heat map (Fig. 4). With exception of brown seaweeds, the green and red seaweeds had significantly different and unique bacterial community composition at genera level (P < 0.05). For instance, the green seaweed (SW1) had *Carnobacterium, Paracoccus, Tepidibacter, Sulfurimonas, Fusobacterium, Roseomonas* and *Rhodobacter* spp. as the dominant bacterial genera while *Desulfotalea, Marinomonas, Shewanella, Thallasomonas, Fluvicola, Polaribacter, Sulfospirillum, Ruegeria, Methylobacterium, Tenacibaculum* and *Fusibacter* spp., dominated SW5. The full epiphytic bacterial genus distribution on different seaweed samples is presented in Fig. 4.

### Functional predictions and interaction analysis

**Figure 5:**
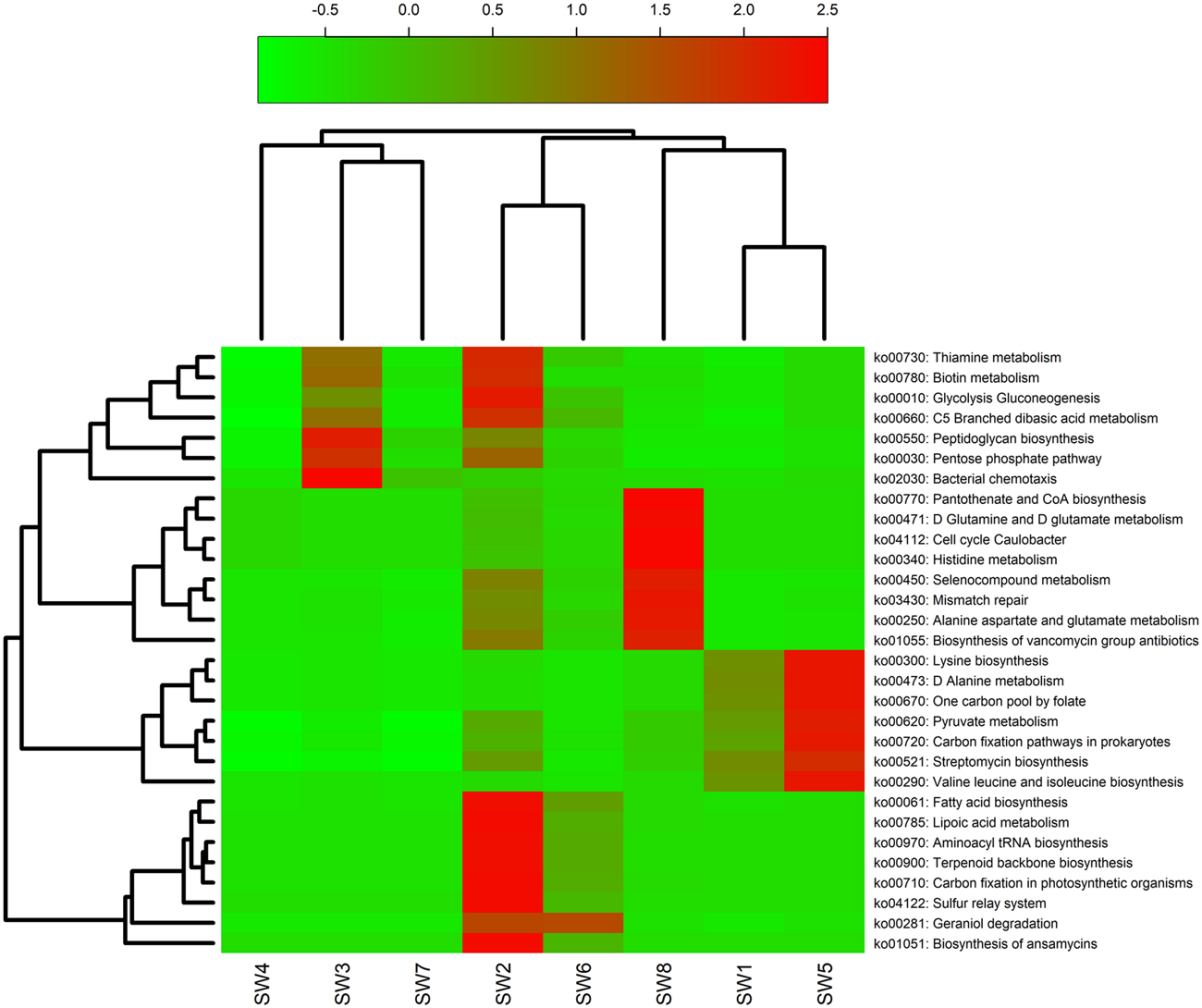
Functional prediction for epiphytic bacterial populations on collected seaweed surfaces.

Functional capabilities of epiphytic bacteria in seaweed samples were predicted using PICRUSt analysis. The obtained NSTI (nearest sequenced taxon index) value was low (0.06–0.18; S. Table 3) indicating that the prediction was accurate as previously described by Langille *et al*.^24^. Breakdown of all predicted metagenomes into KEGG pathways showed that sample SW3 had the highest number of KEGG pathways (279) while sample SW7 had the least KEGG pathways (248). The 30 most abundant predicted pathways have been presented in Fig. 5. The genes most associated with metabolic pathways were for thiamine metabolism (ko00730), biotin metabolism (ko00780), histidine metabolism (ko00340), alanine aspartate and glutamate metabolism (ko00250), D-glutamine and D-glutamate metabolism (ko00471), Selenocompound metabolism (ko00450), D-alanine metabolism (ko00473), pyruvate metabolism (ko00620) and lipoic acid metabolism (ko00785). A relatively higher abundance of biosynthetic genes for peptidoglycan biosynthesis (ko00550), lysine biosynthesis (ko00300), fatty acid biosynthesis (ko00061), aminoacyl tRNA biosynthesis (ko00970), terpenoids biosynthesis (ko00900) and biosynthesis of streptomycin (ko00521), vancomycin (ko01055) and ansamysins (ko01051) antibiotics was also detected. Other important identified functional interactions included bacterial chemotaxis (ko02030), mismatch repair (ko03430), sulfur relay system (ko04122) and geraniol degradation (ko00281), as shown in Fig. 5.

**Figure 6:**
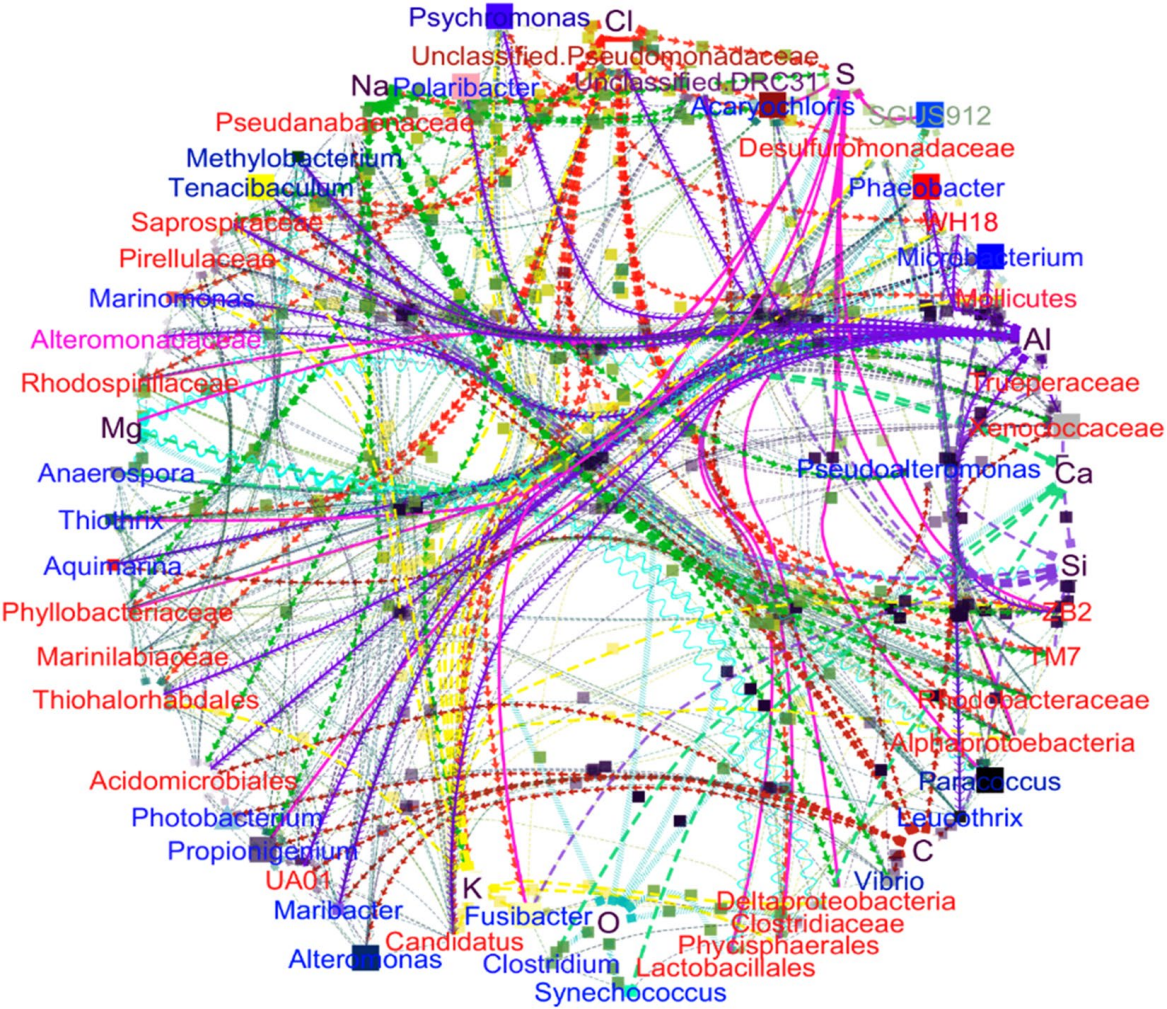
Co-network associations between surface elements and epiphytic bacterial communities on seaweed surfaces.

In order to identify the structural association between OTUs and surface elemental composition of the seaweeds, ten elements (Al, C, Na, Cl, O, K, Ca, Mg, S, and Si) obtained from the EDX results were included along with the top 50 OTUs for co-network interaction analysis. Figure 6 shows the comprehensive association and network of elemental-microbes interaction. The resultant characterized by a topology of 60 nodes and 726 edges, with an average degree, eccentricity, topological co-efficient, radiality and average path length of 25.034, 3.02, 0.489, 0.824 and 1.702, respectively. Results revealed that elements on the surface of the seaweeds influenced unique interactions of bacterial communities. For instance, elemental sulphur had strong interactions with the genera *Acaryochloris*, *Thiothrix, Propionigenium, Fusibacter, Paracoccus*, and some unclassified members of *Deltaproteobacteria*, *Alphaproteobacteria* and *Rhodospirillaceae* groups (S. Fig. 4). Members of the genera *Vibrio*, *Pseudoalteromonas* and *Tenacibaculum* also showed strong inclination towards Na, while the genus *Anaerospora, Clostridium* and *Synechococcus* tended to cluster towards regions of high Ca concentration. Seaweed surfaces with high in C and Cl showed strong interaction with *Alteromonas, Maribacter, Photobacterium, Aquimarina*, *Vibrio, Leucothrix, Marinomonas, Polaribacter, Psychromonas* and *Tenacibaculum*. Further, the identified elements interacted with each other to form complex elements, which also formed networks with bacterial communities on seaweed surfaces.

## Discussion

Macroalgae (Seaweeds) are known to harbour diverse bacterial symbionts on their surfaces, which contribute to morphological development and defence mechanisms^12^. These epiphytic bacterial communities are highly cosmopolitan, and remarkably differ from the free-living bacteria in their surrounding water environment^20^. Nevertheless, the understanding of the epiphytic bacterial diversity and complex interactions with their hosts is still in its infancy. Southern Africa has an extremely rich and diverse seaweed flora with a wide variety of marine habitats^25^. In the past few decades, seaweed-associated epiphytic bacterial communities have been significantly explored in different regions using culture dependent studies and community fingerprinting analysis^7,11,26–28^. However, such studies have been limited in South Africa. Recent advances in molecular tools like next generation sequencing approaches have been focused on exploring microbial diversity at the functional level, often starting with marine samples^13^. In this study, seaweed-associated epiphytic bacterial communities from the intertidal zone were explored using high-throughput sequencing analysis, and the predicted functions and interactions between bacterial communities and seaweed surface adsorbed chemical elements were demonstrated.

While earlier studies report that the compositions of seaweed microbiota are generally host specific^19,20^, our findings revealed that phylum level composition of epiphytic bacterial communities on different seaweed samples were almost similar (Fig. 1). However, seaweed-to-seaweed bacterial community composition was significantly different at the genus level (Figs. 3 and 4). Collectively, *Proteobacteria, Bacteroidetes* and *Firmicutes* were the three core bacterial phyla recorded across all seaweed samples. Similar to these results, *Proteobacteria* has also been observed to be most dominant in other related studies^20,21,29^. Bacteria in the phylum *Proteobacteria* are generally environmentally ubiquitous due to, among other factors, their capabilities to facilitate surface colonization and biofilm formation^30^. Members of the phyla *Proteobacteria, Bacteroidetes* and *Firmicutes* have been found to be abundantly distributed in environments with extreme pH, oligotrophic environments, metal-rich environments^31^ and in seawater^32^. Abundance of epiphytic bacteria belonging to the phyla *Bacteroidetes* and *Firmicutes* has been reported in different green and red seaweeds, such as *Bryopsis hypnoides, Bryopsis pennata, Caulerpa racemosa, Caulerpa cylindracea, Ulva intestinalis, Monostroma hariotii*^20,33–35^. In terms of metabolism*, Bacteroidetes* bacteria are well known biopolymer degraders, which allows the growth of colonizing bacteria by providing an aerobic environment within the surface biofilm^36^. Brown seaweed samples were dominated by bacteria of the phylum *Fusobacteria*, followed by *Proteobacteria*, a trend which has not been reported elsewhere. Previous studies have reported the same seaweed (*Sargassum* sp.) to be dominated by *Firmicutes* and *Planctomycetes* bacteria^19,37^. Consistent with findings of this study, bacterial phyla such as *Cyanobacteria, Planctomycetes, Actinobacteria* and *Verrucomicrobia* have previously been reported to be widespread in different seaweeds^19,20,28^, suggesting that the members of these phyla play an important role in different functional traits.

The finding that *Fusobacteriaceae* as the dominant epiphytic bacterial family in brown seaweed samples (*Sargassum incisifolium* and *Sargassum obovatum*) is consistent with those of Miura *et al*.^38^ on a marine brown algae, *Saccharina japonica*. Some important bacterial genera under this family include *Ilyobacter* and *Propionigenium* spp. These genera inhabit marine environments^39,40^ and play an important role in the seaweed’s ability to metabolise carbohydrates to produce organic acids^38^. Microbe-element inter-connections analysis (S. Fig. 5) showed that epiphytic bacterial genera such as *Leucothrix, Alteromonas, Aquimarina, Propionigenium, Maribacter, Photobacterium, Pseudoalteromonas, Tenacibaculum* and *Xenococcaceae* may play a major role in the marine carbon cycle by utilizing organic carbon sources released from the seaweeds, a same phenomenon also observed by Bengtsson *et al*.^41^ and Tao *et al*.^42^. Seaweeds are thought to provide substrate thereby triggering chemotaxis in bacteria that are capable of metabolising algal exudates^5^. Distribution of bacteria under the genera *Clostridium, Anaerospora* and *Synechococcus* strongly interacted with calcium (S. Fig. 6). Bacteria within these genera are postulated to play an important role in the formation of calcium-phosphate minerals, as supported by the common occurrence of fossil microbes in intertidal rocks^43^. These examples illustrate the possible involvement of epiphytic bacterial communities as players in marine element mobilization, which could provide an important window for understanding processes like mineral weathering, biomineralization, bioremediation and biofouling.

Other brown seaweed associated epiphytic bacterial families identified in this study included *Psychromonadaceae*, *Flavobacteriaceae* and *Vibrionaceae*. The identified dominant genera under these families included *Psychromonas, Aquimarina* and *Vibrio* spp. respectively. Members of genus *Psychromonas* are important producers of alginate lysases^44^. Alginate is a polymeric substance that constitutes up to 50% of the brown seaweed cell wall and intracellular material. Alginate lysases break down the alginate and have potential to be used as biotechnological tools for generating bio-active products. There are conflicting reports as to whether the presence of epiphytic *Vibrio* spp. on brown seaweed leaf surfaces is of net beneficial or detrimental effects to the host. Some studies report that epiphytic bacteria of the genus *Vibrio* produce antimicrobial molecules^35^ to protect the host against fouling and potentially pathogenic microorganisms since marine algae have no immune system of their own^45^. Other reports, however, report that some species of *Vibrio* are opportunistic pathogens, might cause disease to the host^12^.

The genus *Aquimarina*, which was first reported on the surface of brown algae in this study, has previously been associated only with red algae^46^. However, its biological or ecological functions are yet to be explored. Among the red and green seaweed samples studied, each species was associated with different epiphytic bacterial families as shown in Fig. 2. This is consistent with the findings of del Olmo *et al*.^28^ who reported considerable variation among the epiphytic bacteria associated with different seaweed species. Bacteria of the genus *Marinifilum*, belonging to the family *Marinifilaceae*, which were dominant in seaweed SW3 and SW8 have previously been reported as members of a sulphate-reducing consortium in anoxic water, which play a significant role in sulphur transformation^47^. However, no direct interaction was observed between *Marinifilum* and sulphur in this study (S. Fig. 4). Sulfate-reducing epiphytic bacteria under the families *Desulfovibrio, Sulfurimonas, Sulfospirillum and Desulfotalea* were, however, recorded in both red and green seaweeds (Figs. 3 and 4). Intertidal zones are rich in sulfur, considerable amounts of which are deposited on seaweed surfaces. Therefore, microbes utilizing sulfur play a major role in energy production and maintenance of the microbial communities^48^. Bacteria under *Fusibacter, Thothrix, Parococcus, Phyllobacterium, Deltaproteobacteria and, Propionigenium* exhibited a strong interaction with sulphur in this study (S. Fig. 4), reflecting their possible involvement in sulphur oxidation and reduction on the surface of seaweeds.

Seaweeds biomass possess ability to bioaccumulate trace metal ions from the aqueous solution dependent on algal species, and its surface morphology and efficiency of forming coordination complexes with specialized transport ligands in their outer membranes. Several studies have also highlighted the relationship between spatial and temporal differences in trace element concentrations and speciation with the functional and phylogenetic diversity of marine prokaryotes^49–52^. This is partly attributed to the key role bacterial communities play in the biogeochemical cycling of the bio-essential metals, that also simultaneously control in part the growth of marine bacteria and their cycling of major nutrients like C and N^52^. In bacteria, trace metals may influence metabolism primarily as a consequence of the role of these metals (Fe, Mn, Zn, Cu, Co, Mo, Ni) as essential cofactors in metalloenzymes. However, at elevated levels these metals become toxic and the resultant heavy metal stress may induce changes in the bacterial community composition. In polluted coastal marine environments, studies have reported increase in the abundance in bacteria from the *Alteromonadales* order, whose members are metal-resistant and/or capable of binding Cu^2+^ and Zn^2+^ cations to reduce their toxicity, in epibionts associated with invasive seaweed *Asparagopsis taxiformis*^53^. Though no trace elements were reported in this study, overall abundance/presence of the order *Alteromonadales* in all seaweed was very low if not present. In agreement with these finding, proportion of predicted metabolic functions generally associated with heavy metal stress response and resistance to toxic compounds such induction of antioxidants, enzymes such as glutaredoxins, synthesis of glutathione and alkaloids^16,54^ were also very low (Fig. 5). Collectively, these findings suggest that trace element stress does not play an important role in shaping the bacterial community composition of epibionts associated with seaweeds habiting the relative pristine conditions of Mission Rocks, South Africa.

There is an emerging consensus that the bacterial community composition on macroalgae is mainly driven by functional genes rather than taxonomic or phylogenetic composition^16,29,55^, but in concert with the microenvironment established by the physiological and biochemical properties of the algal host^53^. However, the role of functional genes underlying microbial community assembly on seaweed surfaces is still a poorly understood subject^8^. In a bid to gain insight in this area, this study used Phylogenetic Investigation of Communities by Reconstruction of Unobserved States (PICRUSt) to analyse and predict the functional capabilities of epiphytic bacterial communities of different seaweeds. Phylogenetically, the bacterial functional profiles of all green seaweeds (SW1, SW5 and SW8) were more closely related compared to those of brown and red seaweeds (Fig. 5). Seaweed proteins contain large fractions of essential amino acids including glycine, alanine, arginine, proline, glutamic, and aspartic acids^56^. This explains why there was higher abundance of bacterial genes associated with amino acid metabolism in this study. The presence of genes associated with the metabolism of alanine, aspartate, and glutamate explains the bacteria’s dependance on algae-provided amino acids as an adaptive mechanism of epiphytic bacteria in seaweeds. Some biosynthetic genes were also identified in this study, including the genes for the biosynthesis of essential amino acids such as valine, leucine, isoleucine, phenylalanine and tryptophan. Functional genes related to C metabolism, mainly believed to be involved in the mineralization of dissolved organic matter under oligotrophic environment provided by rocky intertidal zone of the study site, were also abundant in all seaweeds (Fig. 5). Similar to our findings, Aires *et al*.^54^ reported higher abundance of bacterial groups and functional genes in *A. taxiformis-*associated microbiota in non-eutrophic pristine waters of Cape Verde island. From ecological perspective, seaweeds may also be exploiting their symbiotic interactions with epiphytic bacteria for protection by relying on the secondary metabolites of epiphytic bacteria as chemical defences as they lack an elaborate *in situ* immune system^5^. This hypothesis is supported by our finding that epiphytic bacteria showed higher abundance of biosynthetic genes related to antibiotic biosynthesis such ansamysins in brown and red seaweeds, and streptomycin and vancomycin group in green seaweeds (Fig. 5). These antibiotics generally elicits antimicrobial activity against many Gram-positive and some Gram-negative bacteria, in addition to having varied antiviral activity towards bacteriophages and pox-viruses^57–59^. Thus their production may play important role in modulating the bacterial community composition by limiting the development and growth of other competing microorganisms whilst protecting the host seaweed against pathogenic invasions^60^. Congruent to our findings, members of *Pseudoalteromonadaceae*, *Pseudomonadaceae*, *Aeromonadaceae*, *Alcaligenaceae*, *Halomonadaceae*, *Hyphomonadaceae*, *Micrococcaceae*, *Streptomycetaceae, Rhodobacteraceae*, *Rhizobiaceae*, and *Erythrobacteraceae* are some bacterial families that have been reported with potential antibiotic activity in the different macroalgal species^53,61^. In addition to antibiotics, production of various bioactive compounds (ranging from haliangicin, violacein, pelagiomycin A, korormicin, macrolactines G and M) by seaweed-associated bacteria exhibiting antifungal, antiprotozoal, antifouling and antibiotic activity^53^, may play an important role in inhibiting or outcompeting other epibionts, and are thus essential in maintaining bacterial diversity and ecological functionality on the algal surface microhabitat^8^. However, it will be more interesting to evaluate conditions under which these antibiotic-like substances are produced, their mode of action and role in inter- and intra-species chemical signalling/quorum sensing, important in perturbation of both taxonomic and functional diversity of bacterial community on algal surface microhabitats.

Besides biosynthesis and metabolism, subtle difference in functional genes responsible for bacterial chemotaxis was observed; such genes being relatively high in red seaweeds compared to green and brown seaweeds. Generally, chemotaxis genes are responsible for chemically mediated interaction between two bacterial groups within the same environmental niche. These are play vital role in co-ordinating movement, biofilm formation, stress resistance and secondary metabolite production^5^. Some bacterial species produce regulatory compounds resembling cytokinins, which may help restore seaweed normal morphology if damage occurs during the tidal movements^8^. However, complexity of microbial systems, coupled with low concentrations of these molecules in water, and their rapid uptake are still challenges making it difficult for in-depth understanding of algal surface microhabitats and ocean functioning. One limitation of current study is that all the inferences are based on predicted functional traits by PICRUSt annotation, that may suffer from inherent inaccuracy in resolving functional biogeography in certain ecosystems^62,63^. Therefore, further deep investigations of chemically mediated interactions between epiphytic bacteria and seaweeds in the context of the aforementioned challenges are likely to reveal new molecules and mechanisms that enable higher organism persistence and functioning despite challenging physicochemical conditions of marine ecosystems.

## Conclusion

In summary, this study provides evidence that different seaweeds from the rocky intertidal zone harbour diverse epiphytic bacterial communities displaying unique metabolic and biosynthetic functions critical for beneficial interactions and adaptability to their ecological niche. While epiphytic bacteria may be important for nutrient biogeochemical cycling and secondary metabolite production, they are greatly influenced by the profile of algal metabolites as well as elemental deposits on seaweed surfaces, which triggers chemotaxis responses with the requisite genes to metabolise those substrates. The bacteria in turn produce chemical secondary metabolites, which defend the host seaweed from pathogenic invasions. Studies using a larger, more diverse seaweed population that are focused on deep metagenomic sequencing and metatrancriptomes of the existing epiphytic microbial diversity are necessary to better describe the symbiotic relationships under natural environmental conditions. Future research should also focus on the identification of metabolic and genetic exchange rather than the taxonomic relationships of the epiphytic bacterial populations to gain an in-depth understanding of their interaction.

## Materials and Methods

### Study area and sample collection

Eight different types of seaweeds samples were collected from intertidal zones of Mission Rocks, Cape Vidal, Leven Point (28°16′37.5″S 32°29′11.5″E), South Africa. Mission Rocks is located in iSimangaliso Wetland Park, a natural habitat of a highly diverse community of marine species. This rocky hotspot is popular for shore fishing at high tide as well as for its rock pools at intertidal zone, which are rich in marine life forms. This rocky zone is still regarded as a pristine natural ecosystem with no external pollutants and is characterised by a dense growth of different seaweeds. In the Mission Rocks ecosystem, the upper, middle and lower littoral zones are dominated with green, brown (with few green algae present) and red algae, respectively, with over 53, 35 and 120 species of green, brown and red algae reported^64,65^. In this study, triplicate samples of eight different species of seaweeds (3 green, 3 red and 2 brown seaweeds were identified on the basis on morphological features) commonly found in the rocky surfaces were collected during low tide in a rectangular area of about 150 m by 50 m (Table 2). Seaweeds attached to rocky surfaces were collected using seawater washed forceps and needles. Seaweed samples were placed in sterile plastic and stored in a portable icebox, and then transferred to the laboratory at the University of South Africa in Johannesburg for further analysis.

**Table 2:** Sample code, Taxonomy, Scientific name and its description of collected seaweeds.

Sample code	Taxon	Scientific name	Description
SW1 (green seaweed)	*Chlorophyta*	*Codium extricatum*	*Codium* is common seaweed in tidal (both high and intertidal) pools - Thick bushy codium with dark green velvety appearance. Branches even in diameter and bifurcated (split in two parts). Several different species of algae may be found between the branches.
SW2 (brown seaweed)	*Phaeophyta*	*Sargassum incisifolium*	Thallus is small (about 15–20 cm high) and found bushy appearance. Spherical shape with ear-like appendages or a crown, sometimes smooth. Found as tight clusters, compressed with serrate margins.
SW3 (red seaweed)	*Rhodophyta*	*Hypnea rosea*	Bright red seaweed can grow up to 17 cm long, comprising irregularly branched axes usually tangled with other algae. It contains numerous short spinous ramuli imparting spiky appearance. The tips are sharply pointed and curved, transparent and rosy pink in colour. Commonly found in Mtwalume, KZN, South Africa.
SW4 (brown seaweed)	*Phaeophyta*	*Sargassum obovatum*	Leaves are spatulate, base cuneate, leathery texture, margin coarsely dentate. Thallus is small (about 10 cm high) and bushy. Appearance in tight clusters, compressed with serrate margins.
SW5 (green seaweed)	*Chlorophyta*	*Valonia utricularis*	Distinct club-like appearance and may grow to as much as two inches in length. Thallus of large (1–35 mm diameter) cells forming cushions a few to 20 cm in diameter. Mainly found attached to hard substrate in cracks, crevices or other protected areas (from predators).
SW6 (red seaweed)	*Rhodophyta*	*Gracilaria corticata*	Seaweed appears like reddish brown to yellowish-brown, terete, firm, and stringy and up to 1 m long, usually found in clumps anchored in rocks and sand.
SW7 (red seaweed)	*Rhodophyta*	*Arthrocardia flabellata*	Thallus appear as pale grey-red, slender, 2–4 cm high, with fronds in small groups with axes sparsely pinnate. Branches divided to form a feather-like pattern. The branch segments are heavily calcified giving it a stiff and tough look and feel. The calcification discourages grazers by making it less edible.
SW8 (green seaweed)	*Chlorophyta*	*Codium lucasii*	Thallus appears very dark green in colour, firm, slippery, applanate, irregularly lobed, 2.5–5 mm thick and up to several cm in diameter, adhering more tightly to substratum (rocky surface). It composed of entwined filaments. Mostly situated in low tide mark to intertidal zones.

### Scanning electron microscopy and EDX analysis

Collected seaweeds were analysed using scanning electron microscopy (SEM). Prior to SEM analysis, seaweed samples were cut into small pieces from the algal thalli, rinsed with distilled water and fixed overnight using Karnovsky’s fixative (8% v/v formaldehyde and 16% *v/v* glutaraldehyde and 0.2 M PBS) at 4 °C. The fixed samples were washed and freeze dried following the descriptions of Selvarajan *et al*.^30^. After lyophilisation, the samples were mounted on metal stubs (aluminium holder) and coated with 10 nm gold using a high-resolution sputter coater. The samples were then examined using a JOEL (JSM-IT 300) scanning electron microscope at an accelerating voltage of 20 kV followed by the SEM. Elemental composition of the seaweeds were determined by energy-dispersive X-ray (EDX) analysis operated at 20 mÅ and 20 kV at the Nano Science Research Unit (University of South Africa, Science Campus, South Africa). The samples were measured from 2° to 40° 2θ, with a scan speed of 0.04°/s.

### DNA extraction and sequencing

Extraction of total microbial DNA from the surface of the each species of collected seaweed samples was performed in triplicates as previously described by Bruker *et al*.^66^. The obtained genomic DNA was then amplified by polymerase chain reaction (PCR) using the universal bacterial primers, 27 F (5′-AGAGTTTGATCMTGGC-3′) and 518 R (5′-GTATTACCGCGGCTGCTGG-3′) targeting the conserved bacterial 16S rRNA gene as described by Ramganesh *et al*.^67^. PCR products were purified using a DNA Clean & Concentrator Kit (ZYMO RESEARCH, Irvin, USA). Prior to the library preparation and sequencing process, triplicate samples of each seaweed species were pooled together and then Illumina sequencing adapters and dual-index barcodes were added to the amplicon targets using full complement of Nextera XT indices (Illumina, Inc. San Diego, CA, USA) through 8 cycle PCR (95 °C for 3 min, 95 °C for 30 s, 55 °C for 30 s, and 72 °C for 30 s, with a final extension at 72 °C for 5 min, then cooling at 4 °C). The resulting PCR product was cleaned again using AMPure XP beads. The fragments size (~630 bp) was validated using Bioanalyzer DNA 1000 chip (Agilent, Santa Clara, CA, USA), and quantified using a fluorometric quantification method (Qubit, USA) that uses dsDNA binding dyes. Dilutions were done based on the quantified DNA using 10 mM Tris Buffer (pH 8.5). 5 µl of diluted DNA was aliquoted from each library and mixed for pooling libraries. The pooled final DNA library (4 nM) was denatured and sequenced on an Illumina Miseq System using paired 300-bp reads to generate high-quality, full-length reads of the V3 and V4 regions by Inqaba Biotechnology (Pretoria, South Africa). Finally, the raw fastq files were obtained after trimming the adapters and primer sequences for further bioinformatics analysis.

### Sequence data analysis

The obtained raw sequence datasets were analysed using Mothur pipeline v.1.40.0 as described by Schloss *et al*.^68^. Sequence reads containing less than 50 nucleotides, reads with more than 2% of ambiguities or 7% of homopolymers were excluded during the course of analysis. Likewise, sequences that belong to the mitochondrial and chloroplast origins were also excluded from the analysis. Chimeric sequences were removed using UCHIME algorithm using the *de novo* method^69^. Non chimeric 16S rRNA reads were then classified to the genus level using the Naïve Bayesian classifier algorithm^70,71^ against the SILVA database version 128^72^. A pairwise distance matrix (Euclidean distance matrix) was created from the curated aligned datasets to group sequences into Operational Taxonomic Units (OTUs) at a sequence similarity of 97% for species level identification. Dominant bacterial OTUs were further subjected to BLAST analysis to compare their identity using the NCBI-BLAST tool^73^. The nucleotide sequence data of the closely identified species for the dominant OTUs were retrieved from the NCBI GenBank, and phylogenetic analysis was done using Molecular Evolutionary Genetic Analysis v6 (MEGA6) software^74^. All the 16S rRNA gene sequences were subjected to multiple sequence alignment using the CLUSTAL_X program. Maximum-Likelihood algorithm was used for the construction of a phylogenetic tree. Bootstrap analysis was performed employing 1,000 replicate data sets in order to evaluate the confidence limits of the branching. Nonparametric diversity indices, including the Shannon–Weaver index and the Chao1 richness estimator were calculated at the genetic distance of 0.03 to measure the diversity of bacterial species among the data sets. The percentage of relative abundance of individual taxa within each community was estimated by comparing the number of sequences assigned to a specific taxon against the number of total sequences obtained for that sample. The identified dominant OTUs at genus level were used to generate a heat map to visualize the variations in seaweed bacterial community structure and distribution.

### Functional prediction analysis

To understand the potential genetic capabilities of the seaweed bacterial communities, the PICRUSt (phylogenetic investigation of communities by reconstruction of unobserved states) software package was used as described by Langille *et al*.^24^. Greengenes (May, 2013 release) was used to classify OTUs and their abundances across the samples was used to infer the functional profiles of the bacterial communities based on a constructed phylogenetic workflow of 16S rRNA marker gene sequences. The abundance of the classified OTUs was first normalised by copy number by dividing each OTU by the known 16S copy number abundance prior to functional predictions. Following the normalisation, prediction was performed by first removing the influence of the 16S marker gene copy numbers in the species genomes and obtaining KEGG Orthology (KO) information and KO abundance corresponding to OTUs. The Nearest Sequenced Taxon Index (NSTI) value was used to validate the reliability of predicted functional and metabolic pathways. The predicted relative abundances of genes associated with the top 30 genes were plotted using heatmap. Differentially abundant pathways were determined by calculating the odds ratio, and analysis of variance on the gene abundance using STAMP^75^. Only differentially abundant pathways and KO’s with a minimum abundance of five were considered. A Benjamini–Hochberg adjusted p-value^76^ was calculated to control the false discovery rate (FDR). KO’s and pathways with odds ratio ≥1 and FDR corrected p-value ≥ 0.05 were considered significantly enriched, while the significantly over-represented bacterial families satisfied an odds ratio of ≥2 and FDR corrected p-value ≥ 0.05.

### Co-network and statistical analysis

To observe the structural association between OTUs and surface elemental composition of the seaweeds, ten elements (Al, C, Na, Cl, O, K, Ca, Mg, S, Si) obtained from the EDX results were included along with the top 50 OTUs. Prior to the network analysis, Spearman’s correlation and statistical significance (p < 0.05) were calculated using Calypso Online Tool^77^. The Cytoscape Software package was used to display the Co-occurrence Network Model^78^, which revealed the statistics of the networks including the number of edges and nodes, network diameter, modularity, clustering coefficient, average degree, and average path length, density and heterogeneity. PAST v.3.20^79^ statistical software was used to calculate community richness and diversity indices. The Microbiome Analyst web application was used to perform one-way analysis of variance (ANOVA), Principle Co-ordinate Analysis (PCoA) and visualization of heatmap^80^.

## Supplementary information

Supplementary Information

## Data Availability

All of the data analysis results obtained during this study are included in this manuscript (and its Supplementary Information files). All the raw datasets from illumina sequencing were deposited at the NCBI database (https://www.ncbi.nlm.nih.gov/) sequence archive (SRA) with submission No PRJNA553359.
