# Supplementary material for: Distribution, Interaction and Functional Profiles of Epiphytic Bacterial Communities from the Rocky Intertidal Seaweeds, South Africa
Source: Sci Rep. 2019 Dec 27;9:19835. doi: 10.1038/s41598-019-56269-2 (PMC6934600; doi:10.1038/s41598-019-56269-2)

**Supplementary Material**

**Distribution, Interaction and Functional Profiles of Epiphytic Bacterial Communities from the Rocky Intertidal Seaweeds, South Africa**

Selvarajan Ramganesh^1^, Sibanda Timothy^2^, Siddarthan Venkatachalam^3^, Henry JO Ogola^1,4^, Chinedu Christopher Obieze ^5^, Titus A Msagati^6^

*^1^Department of Environmental Sciences, College of Agricultural and Environmental Sciences, UNISA, South Africa.*

*^2^Department of Biological Sciences, University of Namibia, Mandume Ndemufayo Ave, Pionierspark, Windhoek, Namibia.*

*^3^Arctic Division, National Centre for Polar and Ocean Research, Vasco-da-gama, Goa, India*

*^4^Centre for Research, Innovation and Technology, Jaramogi Oginga Odinga University of Science and Technology, Bondo, Kenya.*

*^5^Department of Microbiology, University of Port Harcourt, Nigeria*

*^6^Nanotechnology and Water Sustainability Research Unit, College of Science, Engineering and Technology, University of South Africa-Science Campus, Florida, South Africa*

**Corresponding Author**

**Dr Selvarajan Ramganesh**

Dept. of Environmental Sciences

College of Agricultural and Environmental Sciences

University of South Africa – Science Campus

South Africa

Telephone: +27-011-471-2068

Email: ramganesh.presidency@gmail.com

S. Table 1: Percentage of elemental compositions of the collected seaweed surface determined using energy-dispersive X-ray (EDX) analysis

| **Elements** | **SW1** | **SW2** | **SW3** | **SW4** | **SW5** | **SW6** | **SW7** | **SW8** |
| --- | --- | --- | --- | --- | --- | --- | --- | --- |
| **C** | 40.49 | 64.14 | 71.01 | 45.04 | 31.3 | 47.5 | 48.04 | 51.94 |
| **O** | 33.2 | 19.94 | 9.87 | 21.12 | 13.03 | 24.08 | 33.5 | 23.88 |
| **Cl** | 8.66 | 5.88 | 9.93 | 16.06 | 27.05 | 8.16 | 3.45 | 8.92 |
| **Na** | 8.39 | 4.36 | 7.85 | 13.5 | 9.42 | 5.45 | 2.05 | 4.25 |
| **S** | 4.45 | 0.88 | 1.02 | 0.36 | 2.53 | 1.15 | 0.89 | 1.44 |
| **Ca** | 2.69 | 1.9 | 0.12 | 1.05 | 1.5 | 12.42 | 11 | 1.45 |
| **Mg** | 1.6 | 0.74 | 0.02 | 0.01 | 0.5 | 1.17 | 1.01 | 1.53 |
| **Si** | 0.44 | 0 | 0.05 | 0.02 | 0.3 | 0.04 | 0.01 | 6.17 |
| **Al** | 0.06 | 0.28 | 0.06 | 0.01 | 0.1 | 0.02 | 0.04 | 0.32 |
| **K** | 0.02 | 1.88 | 0.07 | 2.83 | 14.27 | 0.01 | 0.01 | 0.1 |
| **Total** | 100 | 100 | 100 | 100 | 100 | 100 | 100 | 100 |

S. Table 2: Relative abundance of minor epiphytic bacterial phyla observed in the collected seaweed samples

| **Minor Phyla** | **SW1** | **SW2** | **SW3** | **SW4** | **SW5** | **SW6** | **SW7** | **SW8** |
| --- | --- | --- | --- | --- | --- | --- | --- | --- |
| *Parcubacteria* | 0.359 | 0.018 | 0.078 | 0.084 | 2.048 | 0.090 | 0.000 | 2.093 |
| *Chloroflexi* | 0.294 | 0.166 | 0.489 | 3.693 | 0.405 | 0.008 | 0.020 | 0.734 |
| *Bacteria_unclassified* | 0.212 | 0.300 | 0.419 | 5.177 | 0.996 | 0.553 | 1.097 | 0.438 |
| *Acidobacteria* | 0.147 | 0.000 | 0.000 | 0.000 | 0.043 | 0.000 | 0.000 | 0.012 |
| *SBR1093* | 0.082 | 0.000 | 0.000 | 0.000 | 0.057 | 0.000 | 0.000 | 0.005 |
| *SR1_(Absconditabacteria)* | 0.049 | 0.000 | 0.000 | 0.000 | 0.000 | 0.008 | 0.000 | 0.022 |
| *Gemmatimonadetes* | 0.033 | 0.000 | 0.000 | 0.000 | 0.000 | 0.000 | 0.000 | 0.000 |
| *Gracilibacteria* | 0.016 | 0.013 | 0.295 | 0.000 | 0.220 | 0.000 | 0.000 | 0.267 |
| *Lentisphaerae* | 0.000 | 0.000 | 0.039 | 0.000 | 0.185 | 0.000 | 0.431 | 0.010 |
| *Omnitrophica* | 0.000 | 0.000 | 0.000 | 0.000 | 0.000 | 0.000 | 0.000 | 0.005 |
| *Peregrinibacteria* | 0.000 | 0.000 | 0.031 | 0.000 | 0.128 | 0.000 | 0.000 | 0.090 |
| *Saccharibacteria* | 0.000 | 0.002 | 0.054 | 0.000 | 0.085 | 0.376 | 0.000 | 0.159 |
| *Spirochaetae* | 0.000 | 0.006 | 0.031 | 0.034 | 0.156 | 0.609 | 0.000 | 0.005 |
| *Tenericutes* | 0.000 | 0.000 | 0.000 | 0.000 | 0.014 | 0.000 | 0.000 | 0.000 |
| *WWE3* | 0.000 | 0.000 | 0.000 | 0.000 | 0.000 | 0.000 | 0.000 | 0.049 |

S. Table 3: Number of predicted metagenomes, counts, number of KEGG pathways and NSTI scores for collected seaweed samples

| **Samples** | **KEGG pathways** | **Predicted metagenomes** | **Metagenome counts** | **NSTI score** |
| --- | --- | --- | --- | --- |
| SW7 | 248 | 3476 | 157151 | 0.115 |
| SW4 | 260 | 4066 | 167246 | 0.117 |
| SW1 | 262 | 4429 | 1221706 | 0.114 |
| SW8 | 264 | 4488 | 4022194 | 0.091 |
| SW6 | 266 | 4618 | 3955217 | 0.088 |
| SW2 | 268 | 4754 | 14553314 | 0.180 |
| SW5 | 274 | 4774 | 2642801 | 0.109 |
| SW3 | 279 | 4809 | 1268965 | 0.100 |

S. Figure 1: Scanning electron microscopy micrographs showed the variations in surface morphology of the collected seaweeds


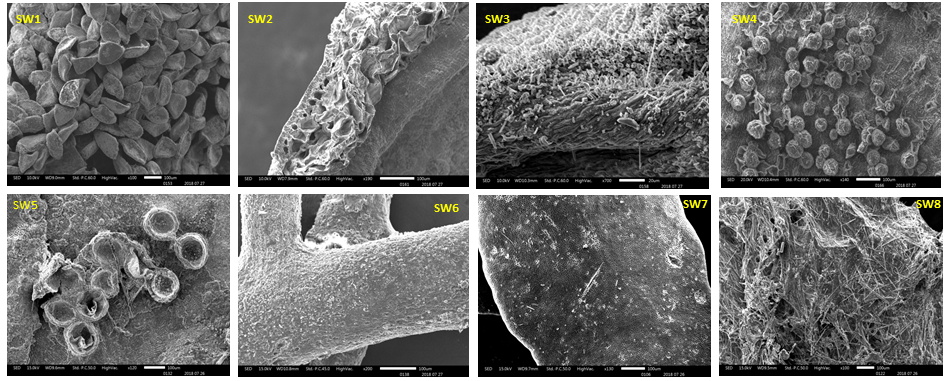


SW1: *Codium extricatum.* SW2: *Sargassum incisifolium* SW3: *Hypnea rosea* SW4: *Sargassum obovatum,* SW5: *Valonia utricularis,* SW6: *Gracilaria corticata*

SW7: *Arthrocardia flabellata* and SW8: *Codium lucasii*

S. Figure 2 : Cluster analysis of epiphytic bacterial community patterns between the collected seaweeds


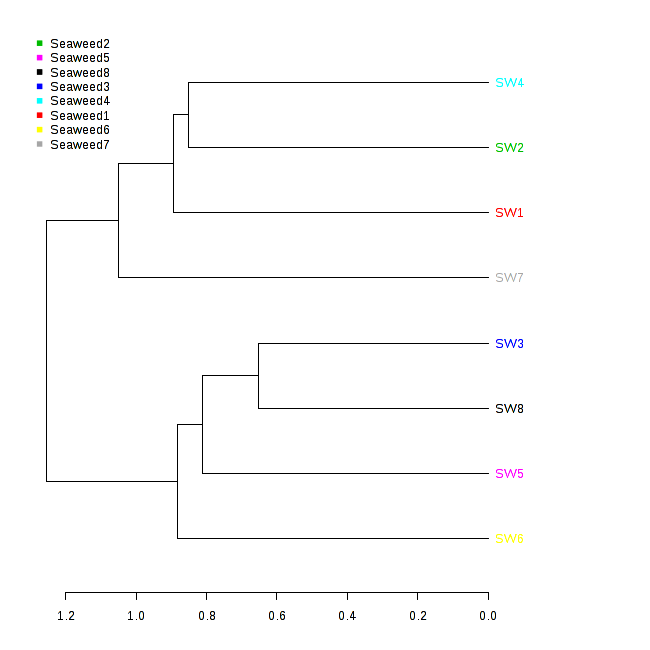


S. Figure 3 : Principal coordinates analysis (PCoA) plot of Bray–Curtis distances for bacterial communities associated with surface of collected seaweeds


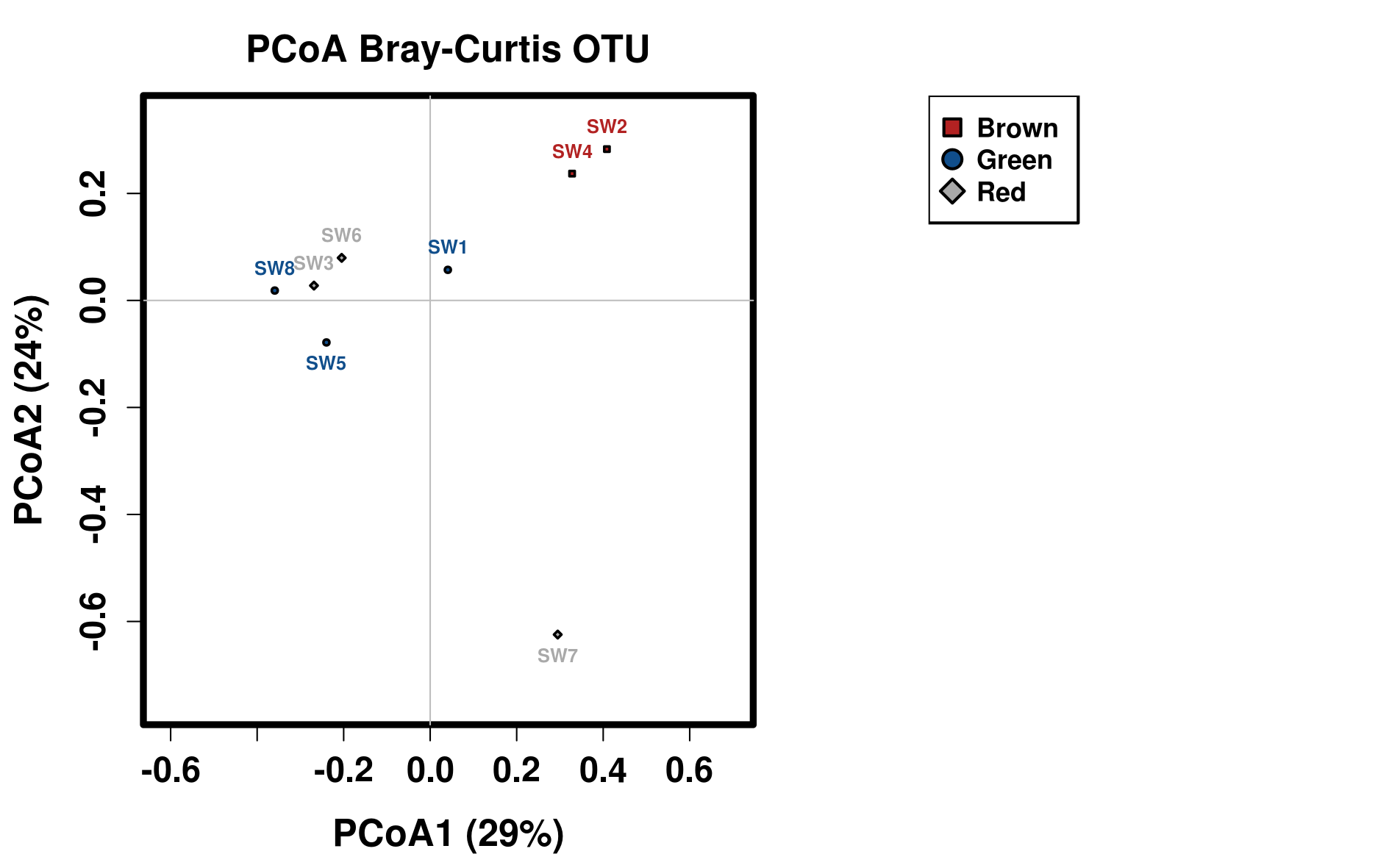


S. Figure 4 : Elemental sulphur interaction with bacterial communities observed on the surface of the seaweeds.


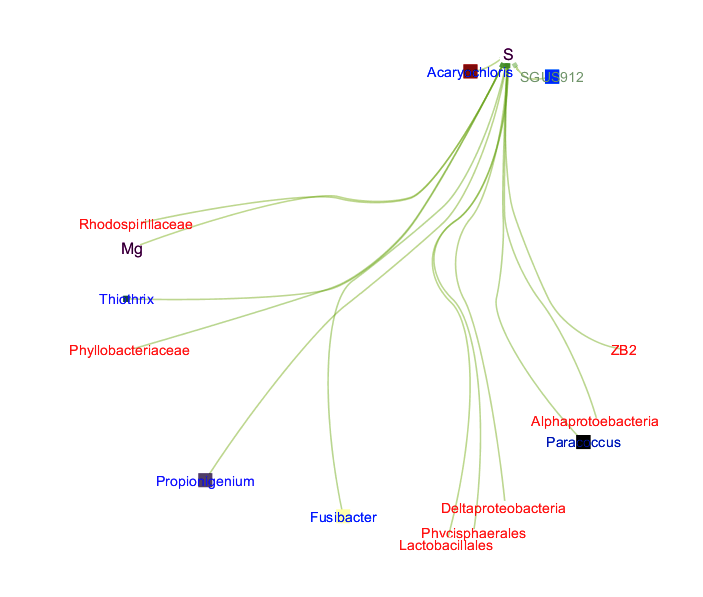


S. Figure 5 : Carbon interaction with bacterial communities observed on the surface of the seaweeds.


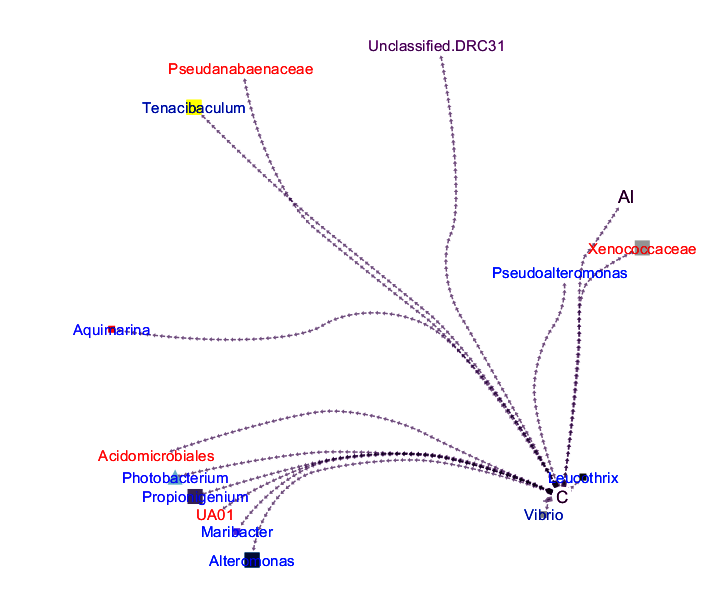


S. Figure 6 : Calcium interaction with bacterial communities observed on the surface of the seaweeds.


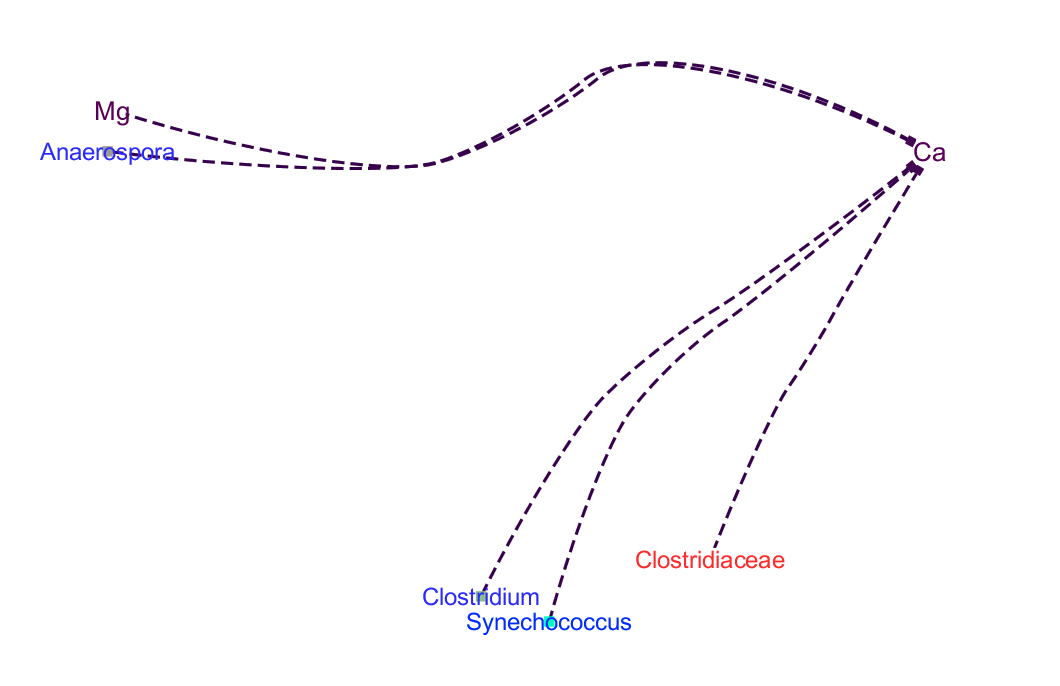

Supplement: Supplementary file 1 — Supplementary Information [file 41598_2019_56269_MOESM1_ESM.docx]
